# Safety Evaluation of the High‐Dose Intake of Heat‐Killed *Lacticaseibacillus paracasei* MCC1849 in Healthy Adults

**DOI:** 10.1002/fsn3.71071

**Published:** 2025-11-17

**Authors:** Natsumi Wada, Kumiko Kato, Satoshi Arai, Soichiro Sato, Noriyuki Iwabuchi, Manabu Nakano, Masahiko Tokushima, Miyuki Tanaka

**Affiliations:** ^1^ Innovative Research Institute, R&D Division, Morinaga Milk Industry Co. Ltd. Zama Japan; ^2^ Maebashi North Hospital Maebashi Japan

**Keywords:** high‐dose intake, *Lacticaseibacillus paracasei*, postbiotics, safety

## Abstract

Heat‐killed *Lacticaseibacillus paracasei* MCC1849 (MCC1849) is a postbiotic used as a functional food ingredient. It has been shown to affect immune regulation both in vivo and in several clinical trials. Based on a previous safety evaluation, the intake of MCC1849 by healthy adults did not raise safety concerns. However, the effects of high‐dose intake have not been investigated. In this study, the safety associated with a high‐dose MCC1849 was evaluated in 20 healthy adults for 4 weeks. The participants received 250 billion cells of MCC1849 per day. Safety evaluations included anthropometric and blood pressure measurements, hematological and biochemical tests, urinalysis, and adverse event monitoring. No clinical differences were detected in anthropometric measurements, blood pressure, or blood and urine parameters between the baseline and post‐intervention measurements. No adverse events associated with MCC1849 intake were observed. In conclusion, an intake of 250 billion cells of MCC1849 per day for 4 weeks was found to be safe in healthy adults. These results suggest that the intake of MCC1849 is safe for use as functional foods, such as for immune‐enhancing effects.

## Graphical abstract

This study evaluated the safety of a high‐dose intake of heat‐killed *Lacticaseibacillus paracasei* MCC1849, a type of postbiotic. No adverse events related to the test food were observed in anthropometric and blood pressure measurements, blood and urine analysis, or medical interviews. These results suggest that the intake of MCC1849 is safe.

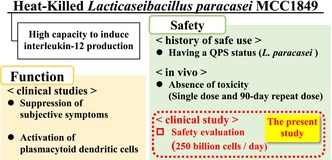

## 1 Introduction

“Cold” is a general term that refers to a mild upper respiratory tract infection caused by various viruses such as coronaviruses, rhinoviruses, and adenoviruses (Heikkinen and Jarvinen 2003). These viruses repeatedly mutate and emerge as novel viruses for sustained transmission (Taubenberger and Kash 2010; Winther 2011). Consequently, tailored treatment for each virus that causes upper respiratory tract infections is difficult, and it is important that the host maintains appropriate immune regulatory functions to prevent these infections.

One approach for maintaining immunity is to consume beneficial food ingredients. These include probiotics, such as lactic acid bacteria (Hill et al. 2014). Furthermore, it has been reported that there are some bacterial strains with immunomodulatory functions in the category “postbiotics,” which are defined as non‐living microorganisms and their components that have health benefits for the host (Salminen et al. 2021).

A type of postbiotic, heat‐killed *Lacticaseibacillus paracasei* MCC1849 (strain Shield, hereafter referred to as “MCC1849”), is known for its immunomodulatory functions. MCC1849 is highly capable of inducing interleukin (IL)‐12 production (Arai, Iwabuchi, et al. 2018). In murine models, oral intake of MCC1849 significantly increased the amount of antigen‐specific immunoglobulin A in the small intestine, serum, and lungs (Arai, Iwabuchi, et al. 2018). In human studies, the intake of 50 billion cells of MCC1849 for 4 weeks significantly increased the expression level of CD86, an indicator of plasmacytoid dendritic cell activation (Kato et al. 2024). In addition, significant reductions in subjective symptoms related to physical conditions were reported when 10–50 billion cells of MCC1849 were consumed for 12–24 weeks (Murata et al. 2018; Sato et al. 2023). The immunomodulatory effects of MCC1849 may be mediated by phagocytosis of plasmacytoid dendritic cells, leading to its activation (Li et al. 2024).

Several evaluations of MCC1849 have indicated a few safety concerns regarding human consumption. In vivo studies, both a single dose of 6000 mg/kg (2.21 × 10^12^ particles/kg) and a repeated dose of 1000 mg/kg/day (3.27 × 10^11^ particles/kg/day) for 90 days, showed no signs of toxicity (Arai, Minami, et al. 2018). In clinical studies, healthy adults ingested 50 billion cells of MCC1849 per day, and no adverse events were associated with MCC1849 (Kato et al. 2024; Sato et al. 2023). MCC1849 can be blended with various foods and supplements that are easy to consume. Therefore, the safety of an overdose must be assessed. Here, we aimed to conduct a single‐arm study to evaluate the safety associated with a high‐dose intake of 250 billion cells of MCC1849 per day for 4 weeks in healthy adults.

## 2 Materials and Methods

### 2.1 Study Design

This open‐label, single‐arm study was conducted in Maebashi City, Gunma Prefecture, Japan, from February to April 2021. The study was approved by the Ethical Committee of the Kobuna Orthopedics Clinic (approval date: February 18, 2021; approval number: MK2102‐1) and registered in the UMIN‐CTR on February 21, 2021 (UMIN000043374). Additionally, this study was conducted in accordance with principles outlined in the Declaration of Helsinki (Fortaleza, revised in 2013) and the Ethical Guidelines for Medical and Health Research Involving Human Subjects (Ministry of Education, Culture, Sports, Science and Technology; Ministry of Health, Labor and Welfare Notification No. 3, 2014, partially revised on February 28, 2017). No protocol changes were made during the study.

### 2.2 Participants

Japanese males and females participated in the study. Before beginning the study, we thoroughly explained the purpose, procedures, and rights of the participants. Then, the written informed consent was obtained from all participants. Participants who provided consent were enrolled according to the following inclusion and exclusion criteria.

The inclusion criteria were: (1) participants aged > 20 and < 65 years, (2) participants who received an adequate explanation of the test objectives and details, who provided consent, who were willing to participate with a good understanding, and who signed the informed consent document. The exclusion criteria were: (1) participants who had a history of serious illness, (2) participants who were under treatment with medication for lifestyle‐related diseases (diabetes, hypertension, dyslipidemia), (3) participants who had digestive tract diseases that affect digestion and absorption, and had a history of gastrointestinal surgery (excluding appendicitis), (4) participants who had a medical history of serious allergy to medicine, food, and pollen, (5) participants who were pregnant or under lactation, or who were expected to be pregnant during the study, (6) participants who were under treatment for or had a history of drug addiction and/or alcoholism, (7) participants who were planning to participate in other clinical studies, and (8) participants who were judged unsuitable for participating in this study by the principal investigator.

### 2.3 Test Food

The test food consisted of a powder containing 250 billion cells of MCC1849 and maltodextrin. The number of MCC1849 was five times the maximum dose used in previous efficacy studies (Kato et al. 2024; Sato et al. 2023). The test foods and MCC1849 were obtained from Morinaga Milk Industry (Tokyo, Japan). Quality assurance procedures confirmed compliance with standards through assessments for bacteria or foreign matter contamination.

### 2.4 Intervention

This study consisted of an intervention period of 4 weeks and a follow‐up period of 2 weeks. Participants received the test food once daily during the intervention period. The intake rate of the test foods was calculated based on the participants' diary records. Participants were instructed not to use any medications, health foods, functional foods, or dietary supplements during the study period. If they had consumed any of these prohibited items, it was recorded in their diary. In addition, participants were instructed to avoid significant lifestyle changes and excessive drinking or eating habits. As this was a single‐arm, open‐label study, no blinding was applied.

### 2.5 Sample Size

The number of participants was set to 20 in a single group to allow for statistical evaluation.

### 2.6 Assessment

Participants visited the clinic for screening at weeks 0, 2, and 4 during the intervention period and at week 6 after the follow‐up period. Changes in physical condition, treatment, and hospital visits were investigated using participants' diaries for 8 weeks, starting from 2 weeks before intake to 2 weeks after intake.

#### 2.6.1 Anthropometric and Blood Pressure (BP) Measurements

Height was recorded exclusively during screening. Body weight (BW), systolic blood pressure (SBP), diastolic blood pressure (DBP), and pulse rate were monitored at every clinic visit. Blood pressure and pulse rate were assessed using a fully automatic blood pressure monitor (TM‐2656VPW; A&D Company Ltd.).

#### 2.6.2 Blood and Urine Analysis

Blood and urine analyses were conducted at the time of the clinic visit. Hematology tests were used to measure the following parameters: white blood cell count (WBC), red blood cell count (RBC), hemoglobin (Hb), hematocrit (Ht), platelet count (PLT), mean corpuscular volume (MCV), mean corpuscular hemoglobin (MCH), mean corpuscular hemoglobin concentration (MCHC), and white blood cell count (proportion of neutrophils, lymphocytes, monocytes, and the percentage of eosinophils and basophils). Biochemical tests measured the following parameters: aspartate aminotransferase (AST), alanine aminotransferase (ALT), lactate dehydrogenase (LD), total bilirubin (TB), alkaline phosphatase (ALP), gamma‐glutamyl transpeptidase (γ‐GT), creatine kinase (CK), fasting blood glucose (FBG), hemoglobin A1c (HbA1c), total cholesterol, LDL‐cholesterol, HDL‐cholesterol, triglycerides (TG), total protein (TP), albumin (ALB), blood urea nitrogen (BUN), creatinine (CRE), uric acid (UA), sodium (Na), chloride (Cl), potassium (K), calcium (Ca), inorganic phosphorus (IP), magnesium (Mg), and serum iron (Fe). The following parameters were measured under urinalysis: urine specific gravity (USG), urine pH (U‐pH), protein (U‐pro), glucose (U‐glu), urobilinogen (U‐uro), bilirubin (U‐bil), ketone bodies (U‐ket), and occult blood reactions (OBR). Hematological, biochemical, and urinalysis tests were conducted by the LSI Medience Corporation (Japan).

### 2.7 Assessment of Adverse Events

Unfavorable or unintended events that occurred during the study period were defined as adverse events. Specifically, the principal investigator evaluated the events based on the participant's condition during the visit, the details in the participant's diary on changes in physical condition, hospital visits, treatment, and the results of blood and urine tests, as well as the situation before intake and the range of variation for each reference value. Adverse events associated with the intake of the test food were determined by the principal investigator.

### 2.8 Statistical Analysis

An independent data manager oversaw all data management. The population for statistical analysis included all participants who consumed the test food at least once. Statistical analyses were conducted using a two‐tailed test, and a significance level was set at *p* < 0.05. The values obtained at week 0 were used as the baseline, and comparisons were made between baseline and each subsequent measurement point. Continuous variables were compared using a paired *t*‐test. Data from urinalysis, except for specific gravity and pH, which are categorical variables, were coded as “0” for within the reference range and “1” for outside the reference range and were compared using McNemar's test. The purpose of this study was not to verify efficacy, but rather to detect risks from a safety perspective. Therefore, β (Type II) error control was prioritized, and multiple comparisons between the measurement points were not performed. Statistical analyses were carried out using IBM SPSS version. 29.0 (IBM Japan Ltd.).

## 3 Results

**FIGURE 1:**
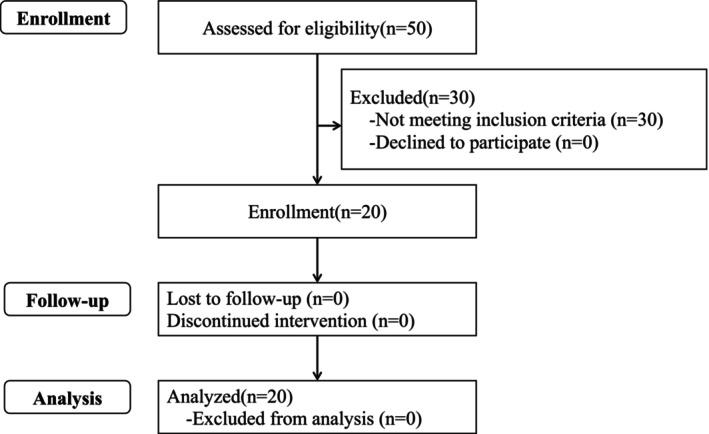
Study flow diagram.

A flow diagram of the study is shown in Figure 1. The participants were recruited between February and March 2021. Of the 50 participants who consented to this study, 20 healthy adults (52.1 ± 7.3 years old, 7 males and 13 females) who met the eligibility criteria were enrolled by the principal investigator. The study was completed by all enrolled participants, who were subsequently included in the analysis. The participants' backgrounds at the time of screening are shown in Table 1.

**TABLE 1:** Participant characteristics at screening.

Characteristics	*n*	Values
Male/Female (*n*)	20	7/13
Age (years) [range]	20	52.1 ± 7.3 [39–63]
Height (cm) [range]	20	163.4 ± 9.4 [150.4–183.7]
BW (kg) [range]	20	60.3 ± 10.6 [45.1–83.3]
BMI (kg/m^2^) [range]	20	22.5 ± 2.6 [18.2–27.5]

*Note:* Data represent number of participants or means ± standard deviations.

Abbreviations: BMI, body mass index; BW, body weight; DBP, diastolic blood pressure; SBP, systolic blood pressure.

### 3.1 Anthropometric and BP Measurements

The results of anthropometric and BP measurements are shown in Table 2. BW (mean difference [MD] 0.22, 95% confidence interval [CI] 0.10 to 0.34 kg, *p* = 0.001) and BMI (MD 0.08, 95% CI 0.02 to 0.14 kg/m^2^, *p* = 0.009) were significantly different between weeks 0 and 2. Body weight (MD 0.31, 95% CI 0.10 to 0.52 kg, *p* = 0.006), BMI (MD 0.11, 95% CI 0.03 to 0.19 kg/m^2^, *p* = 0.008), and pulse rate (MD 2.75, 95% CI 0.06 to 5.44 beats/min, *p* = 0.045) were significantly different between weeks 0 and 4. Pulse rate (MD 2.90, 95% CI 0.32 to 5.48 beats/min, *p* = 0.029) showed a significant difference between weeks 0 and 6.

**TABLE 2:** Summary of anthropometric and blood pressure measurements.

Variables	Reference range	*n*	Week 0	Week 2	Week 4	Week 6
BW (kg)	—	20	60.5 ± 10.5	60.3 ± 10.6**	60.2 ± 10.6**	60.3 ± 10.8
BMI (kg/m^2^)	18.5–24.9	20	22.6 ± 2.6	22.5 ± 2.6**	22.5 ± 2.6**	22.5 ± 2.7
SBP (mmHg)	100–139	20	125.2 ± 10.1	122.9 ± 7.9	123.1 ± 7.3	122.8 ± 6.9
DBP (mmHg)	50–89	20	76.1 ± 7.8	76.2 ± 8.4	75.8 ± 7.5	74.6 ± 7.4
Pulse rate (beats/min)	40–80	20	71.7 ± 6.8	70.3 ± 6.3	69.0 ± 6.2*	68.8 ± 4.6*

*Note:* Data represent means ± standard deviations. *p < 0.05 and **p < 0.01 indicate significant difference compared with week 0 values, according to paired *t*‐test.

Abbreviations: BMI, body mass index; BW, body weight; DBP, diastolic blood pressure; SBP, systolic blood pressure.

### 3.2 Blood and Urine Analysis

Results of the hematological tests are presented in Table 3. The MCHC (MD 0.36, 95% CI 0.09% to 0.63%, *p* = 0.011) was significantly different between weeks 0 and 2. MCV (MD −0.60, 95% CI −1.01 to −0.19 fL, *p* = 0.007), MCH (MD 0.38, 95% CI 0.07 to 0.69 pg, *p* = 0.018), and MCHC (MD 0.61, 95% CI 0.32% to 0.89%, *p* < 0.001) showed significant differences between weeks 0 and 4. No significant differences were observed between weeks 0 and 6.

**TABLE 3:** Summary of hematological examination.

Variables	Reference range	*n*	Week 0	Week 2	Week 4	Week 6
WBC (×10^2^/μL)	33.0–90.0	20	50.6 ± 10.1	52.3 ± 12.1	47.4 ± 12.0	49.2 ± 8.24
RBC (×10^4^/μL)	M: 430–570	7	478.9 ± 46.7	481.7 ± 51.7	479.3 ± 51.2	488.3 ± 45.8
	F: 380–500	13	454.2 ± 34.4	459.2 ± 38.2	454.4 ± 34.9	452.4 ± 37.4
Hb (g/dL)	M: 13.5–17.5	7	14.4 ± 1.2	14.4 ± 1.3	14.3 ± 1.2	14.7 ± 1.2
	F: 11.5–15.0	13	13.6 ± 1.0	13.7 ± 1.0	13.4 ± 1.1	13.6 ± 1.2
Ht (%)	M: 39.7–52.4	7	43.8 ± 3.0	44.3 ± 3.6	44.0 ± 3.3	44.8 ± 3.3
	F: 34.8–45.0	13	42.0 ± 2.5	42.5 ± 2.7	42.3 ± 3.0	42.0 ± 3.3
PLT (×10^4^/μL)	14.0–34.0	20	26.6 ± 6.7	27.5 ± 6.2	26.9 ± 6.7	27.2 ± 5.4
MCV (fL)	85–102	20	92.2 ± 4.1	92.6 ± 4.1	92.8 ± 3.8**	92.6 ± 3.6
MCH (pg)	28.0–34.0	20	30.1 ± 1.5	29.8 ± 1.6	29.7 ± 1.6*	30.0 ± 1.6
MCHC (%)	30.2–35.1	20	32.6 ± 0.8	32.2 ± 0.8*	32.0 ± 0.9**	32.4 ± 0.9
Neut (%)	40.0–75.0	20	57.5 ± 7.1	56.0 ± 8.1	56.8 ± 8.2	57.1 ± 7.5
Lympho (%)	18.0–49.0	20	32.8 ± 7.0	34.0 ± 7.4	33.2 ± 7.7	32.6 ± 6.8
Mono (%)	2.0–10.0	20	5.6 ± 1.4	5.8 ± 1.2	5.7 ± 1.4	5.7 ± 1.3
Eosino (%)	0.0–8.0	20	3.2 ± 1.8	3.4 ± 1.6	3.3 ± 1.9	3.5 ± 2.5
Baso (%)	0.0–2.0	20	0.9 ± 0.4	0.9 ± 0.4	0.9 ± 0.5	1.0 ± 0.5

*Note:* Data represent means ± standard deviations. *p < 0.05 and **p < 0.01 indicate significant difference compared with week 0 values, according to paired *t*‐test.

Abbreviations: Baso, basophils/leukocytes; Eosino, eosinophils/leukocytes; F, female; Hb, hemoglobin; Ht, hematocrit; Lympho, lymphocytes/leukocytes; M, male; MCH, mean corpuscular hemoglobin; MCHC, mean corpuscular hemoglobin concentration; MCV, mean corpuscular volume; Mono, monocytes/leukocytes; Neut, neutrophils/leukocytes; PLT, platelet count; RBC, red blood cell count; WBC, white blood cell count.

The results of the biochemical tests are presented in Table 4. AST (MD 1.95 [0.56, 3.34] U/L, *p* = 0.008), TP (MD 0.24, 95% CI 0.12 to 0.35 g/dL, *p* = 0.001), ALB (MD 0.13, 95% CI 0.05 to 0.21 g/dL, *p* = 0.003), male CRE (MD −0.04, 95% CI −0.07 to 0.00 mg/dL, *p* = 0.038), and Ca (MD 0.15, 95% CI 0.05 to 0.24 mg/dL, *p* = 0.006) showed significant differences between weeks 0 and 2. AST (MD 2.05, 95% CI 0.81 to 3.29 U/L, *p* = 0.003), LD (MD 7.60, 95% CI 0.37 to 14.83 U/L, *p* = 0.040), female γ‐GT (MD 1.08, 95% CI 0.24 to 1.91 U/L, *p* = 0.016), HbA1c (MD −0.15, 95% CI −0.22% to −0.07%, *p* = 0.001), TP (MD 0.19, 95% CI 0.11 to 0.27 g/dL, *p* < 0.001), ALB (MD 0.12, 95% CI 0.05 to 0.19 g/dL, *p* = 0.003), and Ca (MD 0.11, 95% CI 0.02 to 0.19 mg/dL, *p* = 0.018) showed significant differences between weeks 0 and 4. ALB (MD 0.13, 95% CI 0.05 to 0.21 g/dL, *p* = 0.003) showed a significant difference between weeks 0 and 6. The urinalysis results are shown in Tables 5 and 6. No significant differences were observed from 2 weeks before intake to 2 weeks after intake.

**TABLE 4:** Summary of blood biochemistry tests.

Variables	Reference range	*n*	Week 0	Week 2	Week 4	Week 6
AST (U/L)	10–40	20	19.5 ± 3.2	17.5 ± 3.8**	17.4 ± 3.1**	19.2 ± 4.2
ALT (U/L)	5–45	20	16.5 ± 4.9	15.8 ± 5.8	14.9 ± 4.6	17.5 ± 6.3
LD (U/L)	124–222	20	190.5 ± 32.4	183.5 ± 27.3	182.9 ± 27.4*	186.4 ± 30.1
TB (mg/dL)	0.2–1.2	20	0.8 ± 0.2	0.8 ± 0.2	0.8 ± 0.2	0.8 ± 0.2
ALP (U/L)	38–113	20	70.2 ± 24.3	70.9 ± 23.1	68.1 ± 21.9	70.1 ± 21.8
γ‐GT (U/L)	M: ≤ 80	7	30.1 ± 9.7	30.0 ± 11.5	28.4 ± 11.8	30.6 ± 14.1
	F: ≤ 30	13	17.1 ± 6.7	15.8 ± 5.2	16.0 ± 6.1*	17.2 ± 7.4
CK (U/L)	M: 60–270	7	146.3 ± 43.7	130.4 ± 54.0	142.3 ± 59.6	144.6 ± 62.0
	F: 40–150	13	81.0 ± 17.1	79.4 ± 25.9	75.1 ± 15.8	88.8 ± 33.4
FBG (mg/dL)	70–109	20	91.5 ± 5.8	91.5 ± 6.4	91.2 ± 6.0	90.5 ± 5.9
HbA1c (%)	4.6–6.2	20	5.6 ± 0.2	5.6 ± 0.3	5.7 ± 0.2**	5.6 ± 0.3
TC (mg/dL)	120–219	20	215.0 ± 25.9	211.7 ± 19.9	209.6 ± 25.1	212.4 ± 22.0
LDL‐C (mg/dL)	65–139	20	128.8 ± 26.0	123.9 ± 23.1	126.3 ± 23.1	126.1 ± 21.9
HDL‐C (mg/dL)	M: 40–85	7	53.9 ± 15.1	49.9 ± 16.0	51.1 ± 15.0	54.0 ± 16.3
	F: 40–95	13	77.2 ± 10.6	75.5 ± 8.3	77.5 ± 11.8	76.6 ± 10.9
TG (mg/dL)	30–149	20	95.1 ± 47.7	134.6 ± 209.2	95.8 ± 60.7	93.5 ± 48.9
TP (g/dL)	6.7–8.3	20	7.2 ± 0.3	7.0 ± 0.3**	7.0 ± 0.3**	7.1 ± 0.3
ALB (g/dL)	3.8–5.2	20	4.3 ± 0.2	4.2 ± 0.2**	4.2 ± 0.2**	4.2 ± 0.2**
BUN (mg/dL)	8.0–20.0	20	13.0 ± 1.9	13.3 ± 2.3	13.0 ± 2.3	13.6 ± 2.3
CRE (mg/dL)	M: 0.61–1.04	7	0.84 ± 0.12	0.88 ± 0.14*	0.83 ± 0.14	0.90 ± 0.14
	F: 0.47–0.79	13	0.6 ± 0.1	0.6 ± 0.1	0.6 ± 0.1	0.6 ± 0.1
UA (mg/dL)	M: 3.8–7.0	7	5.9 ± 1.0	5.9 ± 0.9	5.6 ± 0.8	6.0 ± 0.8
	F: 2.5–7.0	13	4.4 ± 1.1	4.4 ± 0.9	4.3 ± 1.0	4.4 ± 0.9
Na (mEq/L)	137–147	20	141.6 ± 2.0	141.1 ± 1.7	141.3 ± 1.4	141.2 ± 2.0
Cl (mEq/L)	98–108	20	104.7 ± 1.7	104.5 ± 1.5	104.3 ± 1.7	104.1 ± 2.0
K (mEq/L)	3.5–5.0	20	4.4 ± 0.3	4.3 ± 0.3	4.3 ± 0.3	4.4 ± 0.2
Ca (mg/dL)	8.4–10.4	20	9.5 ± 0.3	9.3 ± 0.4**	9.4 ± 0.3*	9.5 ± 0.2
IP (mg/dL)	2.5–4.5	20	3.6 ± 0.4	3.6 ± 0.5	3.6 ± 0.5	3.7 ± 0.6
Mg (mg/dL)	1.9–2.5	20	2.2 ± 0.1	2.2 ± 0.1	2.2 ± 0.1	2.2 ± 0.1
Fe (μg/dL)	M: 50–200	7	142.0 ± 56.7	103.4 ± 31.9	113.9 ± 25.0	99.7 ± 32.3
	F: 40–180	13	100.1 ± 32.0	88.2 ± 33.4	101.5 ± 40.7	116.9 ± 31.7

*Note:* Data represent means ± standard deviations. *p < 0.05 and **p < 0.01 indicates a significant difference compared with week 0 values according to the paired *t*‐test.

Abbreviations: ALB, albumin; ALP, alkaline phosphatase; ALT, alanine aminotransferase; AST, aspartate transaminase; BUN, blood urea nitrogen; Ca, calcium; CK, creatine kinase; Cl, chloride; CRE, creatinine; F, female; FBG, fasting blood glucose; Fe, serum iron; HbA1c, hemoglobin A1c; HDL‐C, high‐density lipoprotein cholesterol; IP, inorganic phosphorus; K, potassium; LD, lactate dehydrogenase; LDL‐C, low‐density lipoprotein cholesterol; M, male; Mg, magnesium; Na, sodium; TB, total bilirubin; TC, total cholesterol; TG, triglyceride; TP, total protein; UA, uric acid; γ‐GT, γ‐glutamyltransferase.

**TABLE 5:** Summary of urine specific gravity and urine pH.

Variables	Reference range	*n*	Week 0	Week 2	Week 4	Week 6
USG	1.006–1.030	20	1.014 ± 0.007	1.015 ± 0.008	1.013 ± 0.007	1.014 ± 0.008
U‐pH	5.0–7.5	20	6.2 ± 1.0	6.4 ± 0.9	6.2 ± 0.8	6.3 ± 0.9

*Note:* Data represent means ± standard deviations.

Abbreviations: U‐pH, urine pH; USG, urine specific gravity.

**TABLE 6:** Summary of urinalysis results.

Variables	Week	Reference range		*p*
		Within	Outside	
U‐pro	0	20	0	—
	2	19	1	NA
	4	20	0	NA
	6	19	1	NA
U‐glu	0	20	0	—
	2	20	0	NA
	4	20	0	NA
	6	20	0	NA
U‐uro	0	20	0	—
	2	20	0	NA
	4	20	0	NA
	6	20	0	NA
U‐bil	0	20	0	—
	2	20	0	NA
	4	20	0	NA
	6	20	0	NA
U‐ket	0	20	0	—
	2	20	0	NA
	4	20	0	NA
	6	20	0	NA
OBR	0	19	1	—
	2	19	1	1.00
	4	18	2	1.00
	6	19	1	1.00

*Note:* Data represent the number of subjects. *p* values were calculated using McNemar's test.

Abbreviations: MKP, Met‐Lys‐Pro; NA, not applicable; OBR, occult blood reaction; U‐bil, urine bilirubin; U‐glu, urine glucose; U‐ket, urine ketone body; U‐pro, urine protein; U‐uro, urine urobilinogen.

### 3.3 Adverse Events

Two adverse events were reported by two participants during the study period. There were no reports of subjective symptoms in these diaries. The two adverse events observed were high TG and low serum iron levels. Both were mild and transient, and the principal investigator determined that there was no causal relationship with the test food.

## 4 Discussion

In this study, the safety of MCC1849 was evaluated in healthy adults who consumed 250 billion cells per day for 4 weeks. Safety evaluations included anthropometric and BP measurements, hematological and biochemical tests, urinalysis, and medical interviews. No significant changes in urinalysis results were observed compared with the baseline in any of the periods. There were some significant changes in the results of physical, hematological, and biochemical examinations. MCV increased slightly, while MCH and MCHC decreased after MCC1849 intake. Persistent changes in these values can indicate anemia, vitamin B12 deficiency, or folic acid deficiency. A significant but minor reduction was also observed in serum TP and ALB, which are related to protein metabolism, nutrition, and liver function. However, these effects were minimal, and no related symptoms have been observed in long‐term clinical trials or sales monitoring of MCC1849. Importantly, all observed changes were within normal physiological variations or reference ranges; thus, the principal investigator concluded that there were no safety concerns. However, two adverse events were reported: elevated TGs, likely due to a high‐fat meal consumed the day before blood testing, and low serum iron, potentially due to menstruation. All adverse events were judged as accidental and unrelated to the test food by the principal investigator. Therefore, our results showed that there were no safety concerns for healthy adults who consumed 250 billion cells of MCC1849 per day for 4 weeks. Furthermore, although MCC1849 retains immunomodulatory properties, we observed no signs of excessive immune activation even in the case of high‐dose intake, suggesting safety in terms of how it affects the immune response of MCC1849.

MCC1849 belongs to the species *L. paracasei* , which has a history of use in various foods, such as dairy products, fermented and cured meat, and fermented vegetables (EFSA 2007). Owing to its long history of safe use, *L. paracasei* is presumed safe based on the Qualified Presumption of Safety for specific microorganisms, published by the European Food Safety Authority, a public organization (EFSA 2007). Although the Qualified Presumption of Safety list is generally applied to living microorganisms, postbiotics do not have the ability to multiply; thus, they are unlikely to cause bacteremia or fungemia. Therefore, we consider that there is no increase in safety risk owing to heat sterilization. The safety of *L. paracasei* supports the results of this study, which showed that there were no safety concerns, even after 4 weeks of high‐dose intake of MCC1849.

Although this study aimed to evaluate the safety associated with high‐dose MCC1849, it has certain limitations. Firstly, the study participants consisted solely of healthy Japanese adults. Thus, it is unclear whether MCC1849 intake at high doses has an adverse effect on a broader population, including individuals with mild health conditions. Second, as the intervention period was limited to 4 weeks, long‐term effects of high‐dose MCC1849 intake remain unclear. We believe that a comprehensive evaluation based on more information about MCC1849 is required to clarify these limitations.

In conclusion, this study showed that there were no safety concerns associated with healthy adults who consumed 250 billion cells of MCC1849 daily for 4 weeks. Therefore, it is suggested that MCC1849 intake within the range of daily life is safe and can be applied as a postbiotic with immunomodulatory functions.

## Author Contributions

**Natsumi Wada:** formal analysis (supporting); visualization; writing – original draft. **Kumiko Kato:** conceptualization (equal); data curation; formal analThis work was supported by the Morinagaysis (lead); investigation (equal); methodology (equal); writing – review and editing (lead). **Satoshi Arai:** conceptualization (equal); investigation (equal); methodology (equal); writing – review and editing (supporting). **Soichiro Sato:** investigation (equal); writing – review and editing (supporting). **Noriyuki Iwabuchi:** conceptualization (equal); investigation (equal); methodology (equal); writing – review and editing (supporting). **Manabu Nakano:** writing – review and editing (lead). **Masahiko Tokushima:** project administration; writing – review and editing (supporting). **Miyuki Tanaka:** conceptualization (equal); supervision; writing – review and editing (supporting).

## Ethics Statement

The study protocol was approved by the Ethical Committee of the Kobuna Orthopedics Clinic (approval code: MK2102‐1).

## Consent

Written informed consent was obtained from all study participants.

## Conflicts of Interest

This work was supported by the Morinaga Milk Industry Co. Ltd., Tokyo, Japan. N.W., K.K., S.A., S.S., N.I., M.N., and M. Tanaka are employed by the Morinaga Milk Industry Co. Ltd, Tokyo, Japan. The authors declare no conflicts of interest.

## Data Availability

The data that support the findings of this study are available from the corresponding author upon reasonable request.
